# Experiences of social stigma among patients tested positive for COVID-19 and their family members: a qualitative study

**DOI:** 10.1186/s12889-021-11679-8

**Published:** 2021-09-06

**Authors:** Chii-Chii Chew, Xin-Jie Lim, Chee-Tao Chang, Philip Rajan, Nordin Nasir, Wah-Yun Low

**Affiliations:** 1 Clinical Research Centre, Hospital Raja Permaisuri Bainun, Ipoh, Ministry of Health, Malaysia, Bainun, Level 4, Ambulatory Care Centre (ACC), Jalan Raja Ashman Shah, 30450 Ipoh, Perak Malaysia; 2 Otolaryngology Department, Raja Permaisuri Bainun Hospital, Ministry of Health, Ipoh, Malaysia; 3 Hospital Raja Permaisuri Bainun, Ministry of Health, Ipoh, Malaysia; 4 Faculty of Medicine, University of Malaya, Kuala Lumpur, Malaysia

**Keywords:** COVID-19, Social stigma, Patients, Family, Disclosure, Telephone interview

## Abstract

**Background:**

Social stigma against persons infected with COVID-19 is not uncommon. This qualitative study aimed to explore the experience of social stigma among COVID-19 positive patients and their family members.

**Method:**

This cross-sectional study was conducted between April to June 2020 in Malaysia. Patients who have recovered from COVID-19 for at least 1 month and their family members who were tested with negative results, Malaysian and aged 18–65 years old were purposively sampled. Cold call method was employed to recruit patients while their family members were recruited by their recommendations. Telephone interviews were conducted with the participants after obtaining their verbal consent.

**Results:**

A total of 18 participants took part in this study. Three themes emerged from the interviews: (Ι) experience of stigmatization, (ΙΙ) perspective on disease disclosure, and (ΙΙΙ) suggestion on coping and reducing stigma. The participants expressed their experiences of being isolated, labelled, and blamed by the people surrounding them including the health care providers, neighbours, and staff at the service counters. Some respondents expressed their willingness to share their experience with others by emphasizing the importance of taking preventive measure in order to stop the chain of virus transmission and some of them chose to disclose this medical history for official purpose because of fear and lack of understanding among the public. As suggested by the respondents, the approaches in addressing social stigma require the involvement of the government, the public, health care provider, and religious leader.

**Conclusion:**

Individuals recovered from COVID-19 and their families experienced social stigma. Fear and lack of public understanding of the COVID-19 disease were the key factors for non-disclosure. Some expressed their willingness to share their experience as they perceived it as method to increase public awareness and thereby reducing social stigma. Multifaceted approaches with the involvement of multiple parties including the government, non-governmental organization as well as the general public were recommended as important measures to address the issues of social stigma.

## What we already know

- Social stigma against persons infected with coronavirus disease (COVID-19) and their family members resulting from public fear about this newly emerged infectious disease is not uncommon.
- The affected group have been blamed for contracting the disease but refraining from seeking medical care to avoid social stigma and discrimination.
- Social stigma could negatively impact the emotional, mental, and physical well-being of the persons recovered from COVID-19 as well as their family members.

## What this article adds

- Persons recovered from COVID-19 and their family members experienced isolation and labelling and some of them were blamed by the health care workers for contracting this disease.
- The perspectives on disease disclose were ranging from willingness, selective and refuse to disclose. Some of the participants were willing to share their experiences with the aim to stop the chain of this infectious disease while other refused to share their disease contraction history mainly because they perceived that public understanding about this disease remains lacking.
- The participants suggested that each agency including the government, the general public, healthcare professionals, and religious leaders to play their respective roles in the efforts to mitigate stigma.

## Introduction

Coronavirus disease (COVID-19) is a newly emerged infectious disease resulting from the person-to-person spreading of severe acute respiratory syndrome coronavirus 2 (SARS-CoV-2) [1]. One of the impacts in the context of health is social stigma against people who have contracted COVID-19. Social stigma is defined as negative association related to a person or a group of people or places sharing certain characteristics or specific disease [2–5]. Stigma could affect the emotion, mental, and physical well-being of the inflicted groups. Stigmatized people attributed to infectious diseases may experience rejections from partners, families, friends, dismissal from work, and declined quality of health services received, causing alienation, depression, or anxiety [6–8].

While there were limited studies related to social stigma among individual inflicted by COVID-19, their experience could possibly be similar to the persons affected by infectious disease such as HIV/AIDS and tuberculosis. These patients and people living with them often experience social stigma such as discrimination, devaluation, stereotyping, isolation, discrediting, prejudice, humiliation or aggressive attitude [5, 9]. In a study conducted by Kumar et al. (2017) in India, it was found that among the 104 study respondents living with HIV/AIDS, more than half (53.8%) of them experienced stigma [10]. .Similarly, about 50% of study population in Nigeria had discriminated HIV-infected patients and persons caring for them, and perceived that this population should be blamed for bringing the disease into the community [11].

The family members of the affected patients, especially spouses who had lower educational levels, may experience higher level of depression, anxiety and stress due to the stigmatization [12]. In China, more than two third of the family members living with HIV patients anticipated stigmatization from their social network [13]. In the United States, HIV caregiving-related stigma and non-disclosure of caregiving status was associated with more depressive symptoms among the family members [14]. In Malaysia, HIV/AIDS-related stigma was prevalent among the general public in Malaysia. The public perceived that they will be isolated by the community, feel ashamed and will not disclose to others if someone among their family members contracted this infectious disease. This is was mainly due to fear of the lethal and contagion nature of HIV infection [15].

Stigmatized persons are often associated with non-disclosure of disease status [16], avoidance of medical care and non-adherence to treatment to avoid discrimination [17, 18]. This may severely disrupt the efforts to manage any infectious disease outbreak [16]. In South Africa, 80% of the 400 patients with HIV did not feel comfortable to disclose their status [19]. Meanwhile, the majority of HIV/AIDS patients in Malaysia also choose not disclose their disease, whereby their choice is attributed to fear of stigma and discrimination, social consequences and affecting their family emotions negatively [20].

The issue of non-disclosure was commonly reported during the outbreak of COVID-19 and the reasons for this could possibly be similar to the social stigma associated with other infectious diseases including leprosy, influenza, or severe acute respiratory syndrome (SARS) [16, 21]. Those who developed symptoms of COVID-19 or had close contact with COVID-19 patients may hide their contact history and progress to a more severe stage. This may also cause ongoing transmission and difficulties in controlling infectious diseases during an infectious disease outbreak [22]. The Malaysian government advised the people not to hide their medical and travel history from health care workers, as this may pose significant risk of infecting the latter [23].

Destigmatization of infectious diseases is hence important to reduce the rate of non-disclosure and transmission, and reduce discrimination with improvement in disease awareness among the general population [20]. Given the severity of health-related outcomes resulting from stigmatization, this study aimed to explore the experience of patients and their family members in regards to COVID-19 related social stigma, and their suggestions to mitigate this problem, which could serve as an important input for developing strategies to reduce stigmatization.

## Method

Researchers aim to understand how the patients and their family members perceive their personal experience of contracting COVID-19 disease in the context of stigmatization. Hence, a phenomenological approach was used. This approach allows participants to tell, describe and articulate their stories in depth with greater details as they have had lived through with the COVID-19 disease-related experience [24]. This guided the researchers in drafting the topic guide for the interview. The domains include participants’ perspective and understanding of COVID-19, their experience before the patients admitted to the hospital, during their hospitalization and after their discharge, their intention of disclosure, support they needed and suggestions to mitigate stigma.

This was a qualitative study conducted via telephone interview between April and June 2020 at a tertiary COVID-19 referral hospital in the state of Perak in Malaysia. The purposive sampling technique was employed to recruit participants in this study [24]. Participants were categorized into patients and family members. Patients with Malaysian nationality tested positive for COVID-19, aged ranging from 18 to 65 years old, recovered, and discharged home for at least 1 month were included in this study. The inclusion criteria for the patients’ family members were those with Malaysian nationality who were tested negative for COVID-19, between 18 and 65 years old, have been residing with the patients for at least once a week to provide care and support on a regular basis. There is no clear definition of family involvement in residential long-term care; however, most of the study findings have indicated that up to 70% of family members visited their loved one on weekly basis [25, 26]. It is deemed that family members who stayed close to the patients have a higher chance of experiencing the stigmatization. Those who were unable to speak or understand Malay, English or Mandarin language and have intellectual impairment were excluded from this study.

The initial estimated sample size was 10 participants each from the category of patients and the family members. The actual sample size was determined by the point of themes saturation [27]. A semi-structure interview guide was developed based on the published literatures [10–15] and reviewed by the research team - a senior academician, four clinical researchers and the hospital deputy director. Forward and backward translation of English version to Malay and Mandarin version were conducted by a bilingual researcher (CCC). Ethics clearance was obtained from the Malaysian Medical Research Ethics Committee [reference no.: KKM/NIHSEC/P20–939(12)] prior to data collection. The procedure to obtain verbal consent was approved by the ethics committee prior to conducting this study and all participants who agreed to participate were consented. In the process of obtaining informed consent, the content in the participant information sheet were completely conveyed to the participants verbally. They were informed that a copy of the participant information sheet can be posted or emailed to them should they require one; however, none of them had asked for a copy of this information sheet. This study was conducted in accordance with the Malaysian NIH Guidelines for Conducting Research in the MOH Institutions & Facilities [28].

Potential participants were identified through the medical records office and they were contacted through telephone calls by an interviewer (XJL). The interviewer is a medical doctor with 10 years of practice and 3 years of clinical research experience. She was not involved in the treatment of the patients with COVID-19 and did not know the participants in person. All patients were recruited using cold call method [29]. Patients were then asked to recommend a family member who stayed in the same household to participate in this study. The participants were briefed regarding the aims and purposes of this study using the participant information sheet. An in-depth, one-to-one interview with the participants was conducted via telephone call and the conversation was audio recorded. The participants were allowed to select their preferred language and time for the interview. Each interview took 30 min on average.

All the audio-recordings were transcribed verbatim by two researchers (CTC, CCC) at the end of each interview session. The interviews conducted in the Malay language were subsequently translated into the English language by the same researchers. The data were managed using Microsoft® 365 Excel and analyzed via thematic analysis. CTC and CCC were involved in developing code book by performing line-by-line open coding individually. The codes generated were then compiled. The codes were then fine-tuned to generate descriptive themes. Themes generated were constantly compared with the data to confirm the end point of data saturations. The end point was reached when the data collected were sufficient to account for the emerging themes [24, 30]. At the 12th patients, and the 6th family members, there were no more new themes emerged. Saturation point was reached, and the interview stopped with a final sample size of 18 participants. A consensus on the theme and subtheme generated were reached among the researchers.

## Results

Out of 93 telephone call attempts, 20 answered the calls (14 patients and 6 family members), and two patients declined participation due to busy schedules. The median age of patients was 36.5 (IQR: 29) years old and family members was 56.0 (IQR: 18.3). The duration of admission ranged from 10 to 16 days. Most of the participants had tertiary education, professional occupation, and married. The family members were having at least secondary educational level, most were homemakers, and all married (see Table 1 characteristics of participants).

**Table 1:** Characteristics of participants

Characteristics	n (%)
**Patients** (*n* = 12)	
*Age, median (IQR)*	36·5 (29)
*Duration of Hospitalization, median (IQR)*	11 (4)
*Gender*	
Female	6 (50·0)
Male	6 (50·0)
*Ethnicity*	
Malay	10 (83·3)
Chinese	1 (8·3)
Indian	1 (8·3)
*Education level*	
Primary	0 (0·0)
Secondary	1 (8·3)
Pre-University/ Certificate/ Diploma	3 (25·0)
Degree or above	6 (50·0)
Information not provided	2 (16·7)
*Occupation*^a^	
Professional	7 (58·3)
Semi-professional	3 (25·0)
Labourer	1 (8·3)
Retiree	1 (8·3)
*Marital Status*	
Single	2 (16·7)
Married	10 (83·3)
**Family member** (*n* = 6)	
*Age, median (IQR)*	56.0 (18·3)
*Gender*	
Female	3 (50·0)
Male	3 (50·0)
*Ethnicity*	
Malay	6 (100·0)
Chinese	0 (0·0)
Indian	0 (0·0)
*Education level*	
Primary	0 (0·0)
Secondary	2 (33·3)
Pre-University/ Certificate/ Diploma	2 (33·3)
Degree or above	2 (33·3)
*Occupation*^a^	
Professional	1 (16·7)
Semi-professional	1 (16·7)
Labourer	1 (16·7)
Homemaker	3 (50·0)
*Marital Status*	
Single	0 (0·0)
Married	6 (100·0)

^a^Standard occupation classification 2010

A total of three themes and 11 subthemes emerged from the interviews (see Table 2 themes and subthemes generated).

**Table 2:** Themes and subthemes generated

Themes	Subtheme
Experiences of stigmatization	*Isolation*
	*Labelling*
	*Blame*
Perspectives on disease disclosure	*Willing to disclose freely*
	*Selective disclosure*
Suggestions to reduce stigmatization	*Supports by the government*
	*The society and public*
	*Colleagues’ support*
	*Health care providers’ support*
	*Religious support*

### Theme Ι: experiences of stigmatization

Experience of stigmatization among participants began when they were suspected of contracting the disease and then treated as “infective” after recovering from the disease. Some family members who were disease-free have also been treated unfairly.

#### Isolation

Most of the participants felt isolated primarily because of the behavior of the health care professionals once they were suspected of infection. They were handled as COVID-19 positive even without laboratory test results.

*I felt offended when I was treated as if I had been tested positive for COVID-19. Initially, on March 17, they did not act as if the situation was serious, but I had to take food from the outside. I could really see that they didn’t come to my room, and they just contacted me through phone. The sample was taken on March 19, and then on March 23, they came back and took another sample (then) they did not return to my room since then. Once they even failed to offer me tea, so I had to call to say I didn’t have tea, they apologized for overlooking my room because my room was too far away. At first, I felt insulted. I had to express my needs whenever I had to change (my clothes), I had to ask for clothes,* etc.*, and they left the clothes at the door without entering the room. I haven’t even tested positive yet. If they want to wear PPE, they can just wear it, I don’t mind, but they put the clothes on the side of the door as if I’m seriously sick with the virus. (33 years old female patient, finance assistant)*

Isolation by the health care providers happened even after the patients recovered from the infectious disease. This phenomenon has mainly occurred in a government tertiary hospital when participants went to seek medical care.

*As I see that everyone was fearful (with the disease). The nurse was terrified (laughs), the doctors were afraid. The nurse who checked my temperature and blood pressure was not happy, but I can understand (she was in fear). Then I said, “Why are you scared? I have already recovered; I am even more scared.” She was scared and she was sitting far away from me, she didn’t want to be with me for long, (and) she didn’t even want to talk to me. (58 years old female patient, cashier)*

Some of the recovered participants felt socially isolated because the people around them were trying to keep a distance. A few participants noticed that those suspected but not confirmed COVID-19 positive were also isolated by the neighborhood.

*They were isolating [us], they isolate [us]. (57 years old male patient, pensioner)*

*As I have heard that previously there was a suspected case, not confirmed positive or negative yet (and) when the villagers know, everyone has already started to stay away from him, fear of staying closer, and yet (he was) not positive, there was no case at the same location ... (55 years old, wife of patient, housewife)*

#### Labelling

Some of the patients’ family members expressed that they were being labelled as COVID-19 by the neighbors even after recovery.

*Yes, in the end [the neighbor] were fine, not that worried, but we are still being labelled as COVID-19. (42 years old, husband of patient, lecturer)*

Participants who recovered from the disease were still treated as COVID-19 positive patients by the civil servants.

*I told [him] that I was once COVID-19 positive, but now it’s already negative. Then he said it was all right, and he’s going to call back (but) until now he hasn’t called [me] ...I don’t know when I should visit (the service counter for child birth registration) ... maybe they felt that this (previously) positive COVID (people) would infect them, [and then] infect their families. (33 years old female patient, finance assistant)*

A few participants felt offended when their family members were treated unfairly by their neighbors.

*At first, I was angry when the neighbor told her children, who were playing outside the house, to go home early once she saw my mother hang drying the laundry. My mother felt insulted over this event. (28 years old Male patient, sport and youth ministry assistant).*

#### Blame

Some of the recovered participants were blamed by the health care providers for spreading the disease.

*That doctor scolded me, “oh you’ve just returned to your hometown of Penang for your own sake”. I was wondering (what was wrong with going home). My purpose was to visit my family, and I never expected to get sick, and yet he (the doctor) scolded me. (58 years old female patient, cashier)*

### Theme ΙΙ: perspectives on disease disclosure

The willingness of the participants to disclose their history of COVID-19 varied from willing to disclose freely, disclosure upon official purposes and refusal to disclose.

#### Willing to disclose freely

Some of them were willing to tell others about their history as they felt the need to share the experiences with others and to stop the chain of this infectious disease.

*Sure, I’m going to reveal (about my family member infected with the disease) ... as we had experience (it) so we’re going to share that (information). There was no reason to keep this (the disease) as a secret. (34 years old, husband of patient, assistant medical officer)*

*Yes, I am going to tell that I was positive once and they (people) are going to know about this history too. The decision to disclose this history was mainly with the aim of breaking the chain (of infection). (33 years old female patients, finance assistant)*

#### Selective disclosure

The disclosure of being COVID-19 positive was restricted only for official purpose, as the participants were worried that the public or the shopkeepers would be in fear if they revealed their encounter history.

*I’m going to reveal it If it’s for official purpose; however, if we were to tell the neighbors or (the shopkeeper) when we go for shopping, they’ll be scared and won’t let us go to the store. We have been rid of this disease for months, we’d tell our friends if it was necessary, if not, we’d just smile at them. (42 years old, husband of patient, lecturer)*

Most of recovered participants admitted that they will not disclose their medical history mainly because of fear and lack of understanding among the public.

*I prefer to conceal when given a choice as it is very difficult to control other people’s perception and they were fear of COVID-19 when their understanding is very shallow. Instead of explaining to them, we should just keep them in the dark. (35 years old male patient, medical doctor)*

### Theme ΙΙΙ: suggestions to reduce stigmatization

#### Supports by the government

The participants suggested that the relevant government departments should provide assurance to the public that patients recovered from the disease are not contagious to others.

*They may be terrified of us. Even though we are already negative, we have a high probability of re-spreading the disease, which means the virus could be re-activated. The society is very concerned about this and their responses are very important to us. To me, MOH (Malaysian Ministry of Health) or MKN (Malaysian National Security Council) should provide details where we as COVID patients, former COVID patients will not be “contagious” or will not spread the disease, and others will not be affected by the former COVID patient. This means that workers can return to work without any problems as they have recovered from the illness. (31 years old male patient, teacher)*

In addition, some of the participants wanted to file complaints about the issue of isolation, but they were not sure which government agency they should complain to.

*I would like to make a complaint if there were people who isolated us, to whom or to which department I should address my complaint to. (33 years old female patient, finance assistant)*

#### The society and public

While the issues of isolation were common among participants, most of them asserted that they should be treated equally by the public and not by staying away from them.

*(They) should not stay away [from us]. I want to ask them: ‘if people do this (stigmatization) to you because you have an illness, what are your feelings?’ We have to take care of people’s feelings. This wasn’t just about us, it was about others, too. (55 years old, wife of patient, housewife)*

Some of the recovered participants felt that they need to spend more time sharing their experience with the public about disease contact, its effect, hospital stay and the consequences of not practicing preventive measures when they were asked about methods to reduce stigma.

*We need to spend more time talking to people and let them know how we get infected with the disease, what are the symptoms, what are the conditions in the hospital. We should let them know that if we did not take care of our own well-being, practice social distancing, and the risks would have escalated*. *(31 years old male patient, teacher)*

#### Colleagues’ support

One of the recovered participants hoped to maintain an interpersonal relationship and a cheerful place of work just like before the pandemic.

*If possible, work as before COVID-19. If it is possible to create a warmth of human relationships just as before COVID-19, like a happy, friendly (working) environment, and now all this has been diminished, I understand the safety measures at work in which we can’t get together and have to be alone, we all understand that. (38 years old female patients, staff nurse)*

#### Health care providers’ support

Most of them suggested that patient counselling was important to help them cope with this period. The follow-up telephone calls by public hospital psychiatrists were deemed helpful to check on the patients’ condition.

*May be through emotional (support) like what they are doing now. The government psychiatrist they can call check on them, because some may have gone really bad depression, you know all don’t cope it same way right, maybe some was really down until depression and all, so maybe the psychiatrist doctor can help them out by calling them and asking them about their condition, I think they already started doing that.*

*It could be through emotional support as they do. Government psychiatrists may call to check on them because some of them (the patients) were depressed. You know we (patients) don’t all behave the same way, some were depressed, and psychiatrists can help by calling them and asking about their conditions, I think they (psychiatrist) have already started of doing this. (28 years old female patient, medical doctor)*

The participants felt that counselor may be needed for hospital staff who did not contract the disease and that they should be exposed with knowledge on the prevention of stigmatization against persons tested positive for COVID-19.

*Need to involve counselor I think … counselor can offer some guidance to (hospital) workers who are not COVID positive (on) how to prevent stigma (against person inflicted by COVID-19). For instance, people surrounding have to behave normally when interacting with the discharged person. The recovered person might feel weird when everyone gets to know about (the history of) COVID positive and quarantines. Some of my colleagues who were tested negative were quarantined too. They were being teased by my other colleagues for getting 14 days of “free leaves”. They told me that they were not asking for it, they were suffering during quarantine and others who were otherwise healthy have to work more frequent shift especially night duty. (38 years old female patient, staff nurse)*

Some of them believe that counselling from counsellor was important to some patients. The counselling service is perceived as a channel for patients to ask for more information and to receive care from health care providers.

*Counseling is essential but I know it is not for everyone as some may need helps from people outside the hospital. For me, I’ve been good without any support. Many people could never cope without support. They need help and care, such as love. Sometimes we don’t know that patients have other issues... Many of them will not be able to cope and they would be depressed. It would be better if counselling service is available which we can ask for [advice]. I think that (counselor) should show their concern and I’d like some people care about me too. (58 years old female patient, cashier)*

#### Religious support

Participants suggested that a religious teacher would be helpful in coping with a stressful period.

*He [the Ustaz] may help by supporting those of us who are Muslims. Sometimes, we say a predestined test and the Ustaz may help spiritually if anyone is stressed out. (43 years old female patients, nursing manager)*

## Discussion

This is the first research in the Malaysian context, to the best of our knowledge, describing the stigmatization experiences of patients with COVID-19 and their family members. Experience from the time they were suspected of having the disease, seeking care at the hospital until the patient recovered from the disease were explored in the Malaysian context. This study outlined the views of the participants on the experience of stigma and their willingness to disclose their illness. The feedback on how to minimize stigma is a valuable knowledge for health policy makers in designing policies to combat stigma and support societies’ recovery from this pandemic [31].

The experiences of the participants who were isolated, labelled and blamed for having COVID-19 were similar to those affected by HIV / AIDS and tuberculosis as well as the outbreak of SARS in 2003. Such stigma has been correlated with negative impacts, particularly on mental health, and has already become a prominent public health problem [8, 11, 15, 21]. Of concern, patients reported being blamed by health care providers and their suggestions to improve the awareness of healthcare workers on the prevention of stigma imply the problem of stigma in health facilities. Stigma in health facilities is not uncommon and is often associated with denial of care, provision of sub-standard care, physical and verbal abuse by health care providers [32]. In line with the participants’ suggestion, teaching the health care providers about stigma, its manifestations and effect on patients’ health were measures to reduce stigma. Developing the skills of health care providers to work with stigmatized group, engaging them to be in contact with the stigmatized group while delivering intervention to mitigate stigma, and empowering the stigmatized group to improve coping mechanism were approaches in overcoming stigma at health facilities [32].

Some participants preferred to keep their history of COVID-19 confidential. The reasons for the non-disclosure of the medical history were consistent with the perspective of patients with infectious diseases including leprosy and HIV / AIDS, mainly due to insufficient knowledge and concern about contagious nature of the disease among the public [2, 16, 18, 20]. COVID-19, a highly contagious and new infectious disease with many unknown areas, may be a leading source of public misconceptions and misinformation which are the key contributor to stigma [2, 33]. Another reason for their non-disclosure is the concern over the possibility to be banned from carrying out routine tasks such as purchasing groceries, and these concerns were similar to those in the previous study [20]. Public understanding and compassion are important in reducing stigma [11, 34], as some participants have suggested earlier.

Lack of knowledge and misinformation of the infectious diseases are contributing factors to stigma [2, 31, 33, 35]. Reliable information on disease prevention, treatment options, accessibility of health care in plain language should be disseminated in social media by governments, the communities, media, and key influencer (e.g. religious leader) as potential methods for combating stigma [2, 33, 36]. To keep the public abreast of the COVID-19 situation in Malaysia, the Ministry of Health Malaysia (MOHM) produced daily press statement and press conferences, covering statistics of the recovered COVID-19 cases, number of newly confirmed case, number of confirmed cases by states and new death cases. Apart from the disease statistics, health advisory were regularly disseminated, including important preventive measures such as the 3C: to avoid crowded places; confined spaces; and close conversations [37]. Based on our findings, information on social stigmatization towards the public is important to reduce its occurrence. In India, the Ministry of Health and Family Welfare has advised the public not to blame the COVID-19 patients; provide support to the patients and family members and not to disclose the names or identity of those affected or under quarantine on the social media [38]. The Japan Educational Ministry has warned the education boards over the country to stop discrimination and prejudice against workers exposed to higher risks of contracting the novel coronavirus [39]. It is noteworthy that the MOHM has recently added the mental health and psychosocial aspect in COVID-19 to inform the public on the dos and don’ts when interacting with COVID-19 patients or their family members [34].

Interventions to mitigate infectious disease-related stigma often involving multifaceted approaches and requiring the collaboration of stakeholders from different fields including the government, health care providers, the public and key opinion leaders [2]. The Ministry of Health Malaysia is aware of the importance of stigma associated mental health problem among COVID-19 patients. Stigma reduction was advocated as one of the interventions for mental health support to the affected person, the general public and health care workers were recommended not to define the affected persons as COVID-19 and support them with mental and psychological support [34].

In the Guide to Prevent and Addressing Stigma, WHO urged the authorities and media to share sympathetic narratives that humanize the experiences and struggles of the infected patients or family members [2]. As of June 2020, Malaysia has reported a total positive COVID-19 cases of more than 8000 [37]. The authorities and media may consider inviting patients to share their experience of stigmatization after sufficiently anonymizing their identity. Such interventions were consistent with the suggestions made by some of the participants in this research, who were willing to share their experience of exposure, diagnosis and their perceived stigmatization experience in the community. As reported in a previous study, the dissemination of digital stories using social media may be a feasible way to educate the public, reducing fear and anxiety among the affected communities [40].

Various interventions have been found effective to reduce stigmatization by the healthcare providers towards their patients [32]. It was previously reported that a stigma reduction educational session targeting health providers may improve their attitude and reduce stigmatization among HIV patients [40]. In China, the stigma reduction program implemented to disseminate stigma reduction messages by popular opinion leaders to other healthcare providers was found to be effective [41]. A multifaceted approach with the establishment of hospital steering committees, staff training using a stigmatization awareness module and hospital policy development could effectively reduce stigmatization towards HIV patients [42]. The MOHM has developed a comprehensive Guidelines of COVID-19 Management in Malaysia, which covered the clinical management of COVID-19 cases, sampling procedure, methods for social distancing etc. [43]. The Ministry Guideline (Annex 33) also contained the message to reduce personal identification of patients with COVID-19 (such as victims) but recognized them as “people who are recovering from COVID-19” [34]. While a targeted educational approach may be the most effective way to reduce stigmatization, familiarization and training of healthcare providers with the existing MOHM guidelines may be practical with the current limited resources.

There were several limitations in this study. The findings from our single center study cannot be generalized to other health institutions. The study was conducted during the country’s movement control order lockdown period, where most of the work and social activities of the participants remained restricted, and the experience of stigmatization after being discharged from the hospital cannot be described in full context. Future researches could consider exploring the knowledge level and belief about this disease among the public to identify the knowledge gap and misinformation which in turn would help to address the social stigma issue [33].

## Conclusion

Individuals recovered from COVID-19 and their families experienced stigma, including labelling, isolation and blamed for contracting the disease. Fear and lack of public understanding of the COVID-19 disease were the key factors for non-disclosure. Nevertheless, some recovered patients were willing to share their experience as a way to increase public awareness on negative impacts of stigmatization and thereby helping to reduce stigma. The government, the general public, healthcare professionals, and religious leaders played important roles in the efforts to reduce social stigma issues.

## Data Availability

For confidentiality reasons, qualitative data collected for this study cannot be shared.
